# Supplementary material for: Banning new gas boilers as a no-regret mitigation option
Source: Nat Commun. 2025 Jan 2;16:49. doi: 10.1038/s41467-024-55427-z (PMC11696083; doi:10.1038/s41467-024-55427-z)
Supplement: Supplementary file 1 — Supplementary information [file 41467_2024_55427_MOESM1_ESM.pdf]

# Banning new gas boilers as a no-regret mitigation option

Célia Escribe[*,1,2,+] and Lucas Vivier[*,1,3,+]

[1]CIRED-CNRS, 45 bis, Avenue de La Belle Gabrielle, 94736, Nogent sur Marne, France
[2]CMAP, CNRS, Ecole Polytechnique, Institut Polytechnique de Paris, Route de Saclay, Palaiseau, France
[3]ENPC, Ecole des Ponts ParisTech, France
[+]These authors contributed equally.
[*]Corresponding authors: celia.escribe@polytechnique.edu, lucas.vivier@enpc.fr

## Supplementary Items

- Supplementary Tables
  - Supplementary Table 1: Summary of results across policy scenarios in 2050 . 3
  - Supplementary Table 2: Summary of main results in the residential sector in the configuration setting by 2050. . . . . 4
  - Supplementary Table 3: Impact on discount rates on total system cost. . . . . 4
  - Supplementary Table 4: Evolution of CAPEX. . . . . 14
  - Supplementary Table 5: Evolution of Fixed Operation and Maintenance (FOM) costs. . . . . 15
  - Supplementary Table 6: Other constant electricity generation technology parameters. . . . . 15
  - Supplementary Table 7: Low-carbon technologies potential in 2050. . . . . 16
  - Supplementary Table 8: Evolution of biogas potential. . . . . 16
  - Supplementary Table 9: Evolution of storage CAPEX. . . . . 16
  - Supplementary Table 10: Cost of heating system. . . . . 17
  - Supplementary Table 11: List of data sources used in Res-IRF. . . . . 17
- Supplementary Figures
  - Supplementary Figure 1: Ranking of most influential parameters driving the capacity of the current policy scenario to achieve carbon neutrality. . . . . 5
  - Supplementary Figure 2: Simplified diagram of the the overall efficiency of replacing gas boilers with heat pumps and direct electricity. . . . . 6
  - Supplementary Figure 3: Hourly dispatch to meet electricity demand in 2050 over a typical week in January. . . . . 6
  - Supplementary Figure 4: Load profile for electricity and gas heating demands in 2050 over a typical week in January. . . . . 7

– Supplementary Figure 5: Frequency of scenarios based on total system cost. . 7

– Supplementary Figure 6: Average additional annual costs by household group if the ban is implemented. . . . . . . . . . . . . . . . . . . . . . . . . . . . . . . . . . . . . . . . . . . . . . 8

– Supplementary Figure 7: Distribution of income group among landlords in France in 2018. . . . . . . . . . . . . . . . . . . . . . . . . . . . . . . . . . . . . . . . . . . . . . . . . . . . . 8

– Supplementary Figure 8: Cost of households in 2018 including energy cost, heater systems investment cost, and taxes due to subsidies. . . . . . . . . . . . . . . . . . 9

– Supplementary Figure 9: Stock of heating system by household group in 2018. 10

– Supplementary Figure 10: Additional boilers in 2050, if the ban is implemented, in millions of boilers. . . . . . . . . . . . . . . . . . . . . . . . . . . . . . . . . . . . . . . . . . . . . . . . . 11

– Supplementary Figure 11: Additional boilers in 2050 if the ban is implemented, as a proportion of total installed boilers in 2050 by household group. . . . . . . 12

- Supplementary Methods

  – Supplementary Method 1: . . . . . . . . . . . . . . . . . . . . . . . . . . . . . . . . . . . . . . . . . . . . . 13

  – Supplementary Method 2: . . . . . . . . . . . . . . . . . . . . . . . . . . . . . . . . . . . . . . . . . . . . . 14

  – Supplementary Method 3: . . . . . . . . . . . . . . . . . . . . . . . . . . . . . . . . . . . . . . . . . . . . . 17

# Supplementary Tables

Supplementary Table 1: **Summary of results across policy scenarios in 2050.** Values in billion euros are the sum of actual invested values between 2025 and 2050.

| | **Unit** | **Current policy scenario** | **Ban** |
|---|---|---|---|
| Number of heat pumps | Million | 16 | 20 |
| Number of direct electric | Million | 5 | 7 |
| Number of gas boilers | Million | 8 | 0 |
| Number of wood boilers | Million | 4 | 6 |
| Subsidies insulation | B€ | 60 | 59 |
| Subsidies heater | B€ | 47 | 71 |
| Investment heating system | B€ | 321 | 349 |
| Investment insulation | B€ | 136 | 135 |
| Consumption Electricity | TWh | 42 | 61 |
| Consumption Gas | TWh | 59 | 1 |
| Consumption Wood | TWh | 60 | 76 |
| Offshore capacity | GW | 45 | 39 |
| Onshore capacity | GW | 60 | 60 |
| Solar PV capacity | GW | 75 | 69 |
| Nuclear capacity | GW | 29 | 29 |
| Battery capacity | GW | 3 | 0 |
| Peaking plants capacity | GW | 47 | 59 |
| Methanization capacity | GW | 5 | 5 |
| Pyrogazification capacity | GW | 2 | 1 |
| Hydroelectricity capacity | GW | 18 | 18 |
| Offshore production | TWh | 210 | 183 |
| Onshore production | TWh | 171 | 171 |
| Solar PV production | TWh | 107 | 98 |
| Battery production | TWh | 3 | 0 |
| Hydroelectricity production | TWh | 51 | 51 |
| Peaking plants production | TWh | 14 | 32 |
| Nuclear production | TWh | 137 | 170 |
| Methanization production | TWh | 46 | 46 |
| Pyrogazification production | TWh | 19 | 12 |

Notes. PV refers to photovoltaic.

Supplementary Table 2: **Summary of main results in the residential sector in the configuration setting by 2050.**

| | **Current policy** | **Ban** |
|---|---|---|
| Stock (M) | 40 | 40 |
| Surface (M m2) | 3,490 | 3,491 |
| Consumption (TWh) | 183 | 160 |
| Consumption (kWh/m2) | 52 | 46 |
| Consumption PE (TWh) | 238 | 241 |
| Consumption Electricity (TWh) | 43 | 62 |
| Consumption Natural gas (TWh) | 59 | 2 |
| Consumption Wood fuel (TWh) | 60 | 77 |
| Consumption Heating (TWh) | 20 | 20 |
| Energy poverty (M) | 1.3 | 1.1 |
| Emission (MtCO2) | 13 | 3 |
| Stock G (M) | 0.3 | 0.4 |
| Stock F (M) | 0.6 | 0.6 |
| Stock E (M) | 1.1 | 1.1 |
| Stock D (M) | 7.6 | 6.4 |
| Stock C (M) | 11.4 | 9.9 |
| Stock B (M) | 5.7 | 7.2 |
| Stock A (M) | 12.9 | 13.9 |
| Stock Electricity-Heat pump water (M) | 16.6 | 20.9 |
| Stock Electricity-Direct electric (M) | 5.2 | 7.8 |
| Stock Natural gas-Performance boiler (M) | 8.8 | 0.6 |
| Stock Wood fuel-Performance boiler (M) | 4.9 | 6.1 |
| Stock Heating-District heating (M) | 4.1 | 4.1 |
| Health cost (B €) | 1 | 2 |
| Energy expenditures (B €) | 24 | 21 |
| Cumulated Renovation (Thousand households) | 17,736 | 17,297 |
| Cumulated Investment insulation (B €) | 212 | 209 |
| Cumulated Subsidies insulation (B €) | 61 | 61 |
| Cumulated Investment heater (B €) | 310 | 341 |
| Cumulated Subsidies heater (B €) | 49 | 74 |
| Annual average Renovation (Thousand households) | 572 | 558 |
| Annual average Investment insulation (B €) | 6.8 | 6.7 |
| Annual average Subsidies insulation (B €) | 2.0 | 2.0 |
| Annual average Investment heater (B €) | 10.0 | 11.0 |
| Annual average Subsidies heater (B €) | 1.6 | 2.4 |
| Consumption saving (%) | 33% | 41% |
| Emission saving (%) | 70% | 93% |
| Energy poverty reduction (%) | 64% | 71% |

Notes. A, B, C, D, E, F and G correspond to energy performance certificate classification in France. A stands for the best-performing dwellings while G stands for the worst-performing dwellings.

Supplementary Table 3: **Impact on discount rates on total system cost.** Sensitivity analysis across different discount rates of additional total system costs (in B€) when implementing the ban on gas boilers compared to the current policy scenario, in the reference configuration.

| **Discount rate** | **Additional total system costs** |
|---|---|
| 1.0% | 5 |
| 2.0% | 7 |
| 3.2% | 8 |
| 4.0% | 8 |
| 5.0% | 8 |

## Supplementary Figures

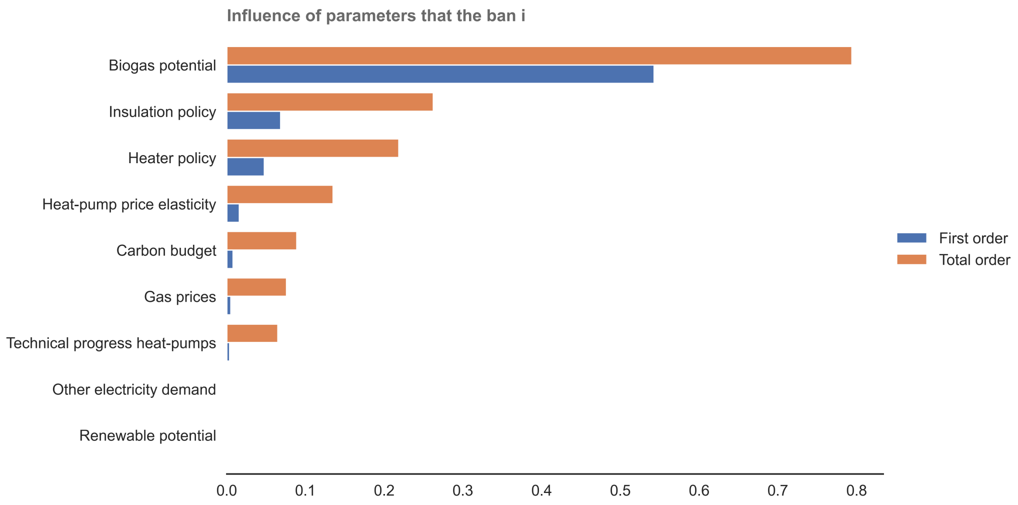


Supplementary Figure 1: **Ranking of most influential parameters driving the capacity of the current policy scenario to achieve carbon neutrality.** First order Sobol indices illustrate the share of variance explained by each uncertainty independently, while total order Sobol indices represent the share of the variance explained by each uncertainty in interaction with other uncertainties.

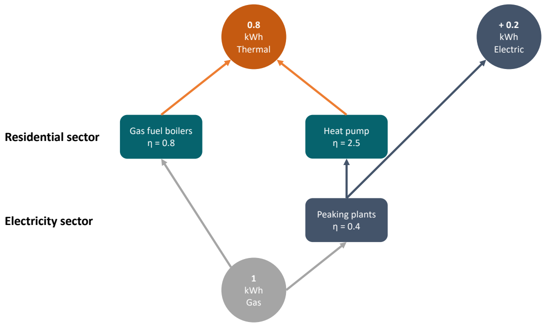


(a)

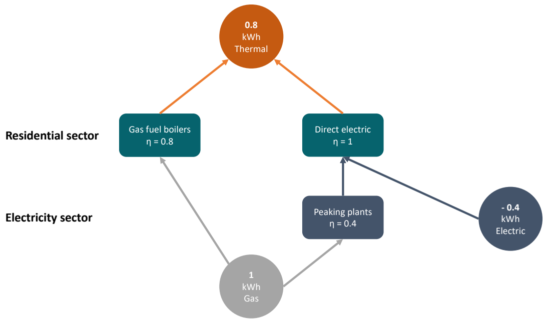


(b)

Supplementary Figure 2: **Simplified diagram of the the overall efficiency of replacing gas boilers with heat pumps and direct electricity.** $\eta$ refers to efficiency of individual system. a. When heat pump is chosen. In the model, heat pump efficiency is not a constant value but varies with outdoor temperature across different days. For simplicity in this figure, we use a value of 2.5, which represents the lower end of the range but still illustrates the higher efficiency of the system. b. When direct electric is chosen.

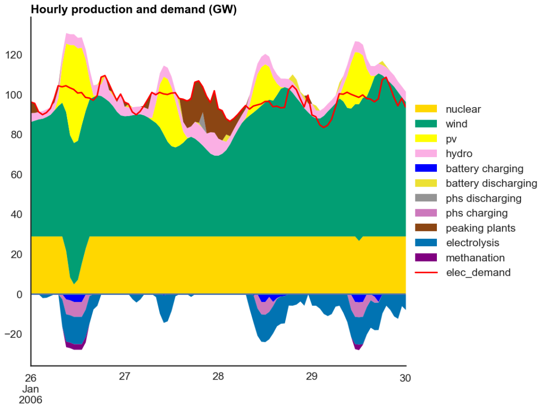


(a)

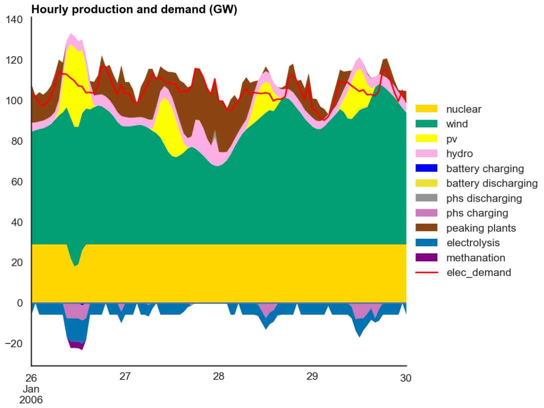


(b)

Supplementary Figure 3: **Hourly dispatch to meet electricity demand in 2050 over a typical week in January.** a. Current policy scenario. b. Ban scenario. PV stands for photovoltaic.

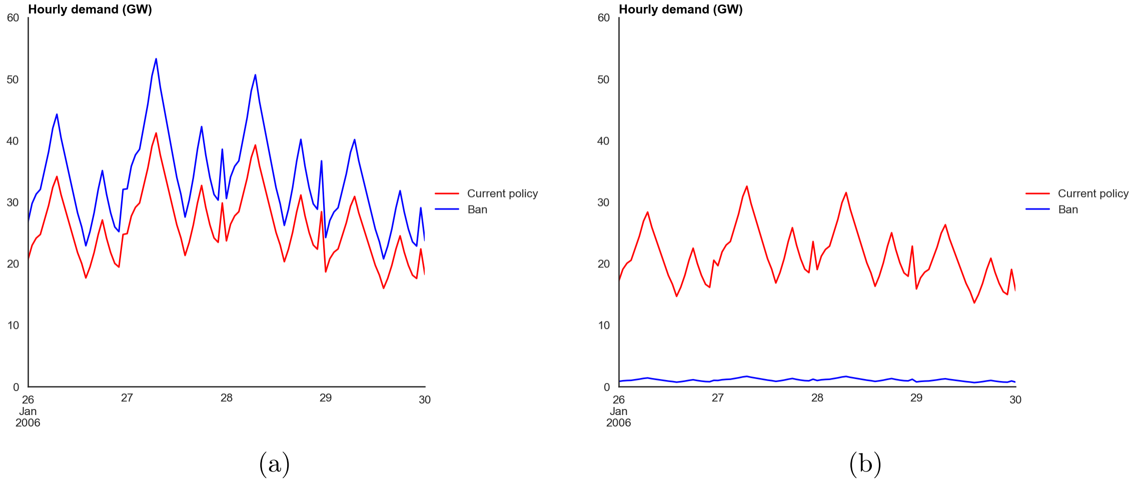


Supplementary Figure 4: **Load profile for electricity and gas heating demands in 2050 over a typical week in January.** a. Electricity demand. b. Gas demand.

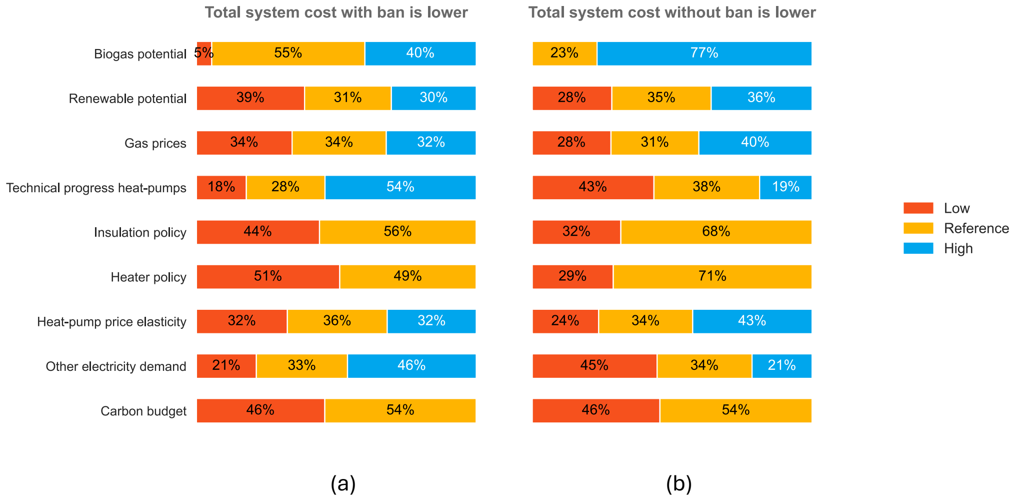


Supplementary Figure 5: **Frequency of scenarios with total system cost lower with the ban (a) and total system cost lower without the ban (b).** Figure identifies the determinants responsible for the higher cost-effectiveness of the current policy scenario compared to the ban. Scenarios exhibiting higher system costs under the ban typically feature high heat pump price elasticity—indicating a strong household investment response to reductions in heat pump prices—substantial biogas potential—suggesting favorable conditions for decarbonizing the residential gas supply—and ambitious insulation policies.

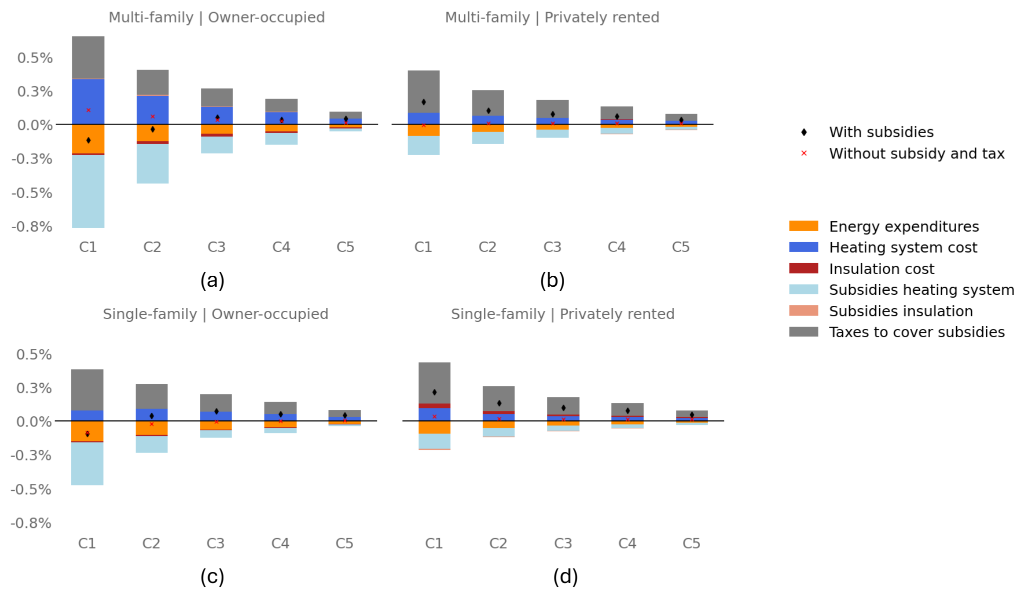


Supplementary Figure 6: **Average additional annual costs by household group if the ban is implemented (%).** a. Owner-occupied in multi-family dwellings. b. Privately rented in multi-family dwellings. c. Owner-occupied in single-family dwellings. d. Privately rented in single-family dwellings. 'C1' means the first income quintile, i.e. very low income, and 'C5' means the last income quintile, i.e. very high income. A negative value means that the ban reduces household expenditure, while a positive value means that the ban increases household expenditure. Total cost is shown net of subsidies and taxes (black diamond) and without including these factors in order to measure the strict effect of the ban before redistribution (red cross).

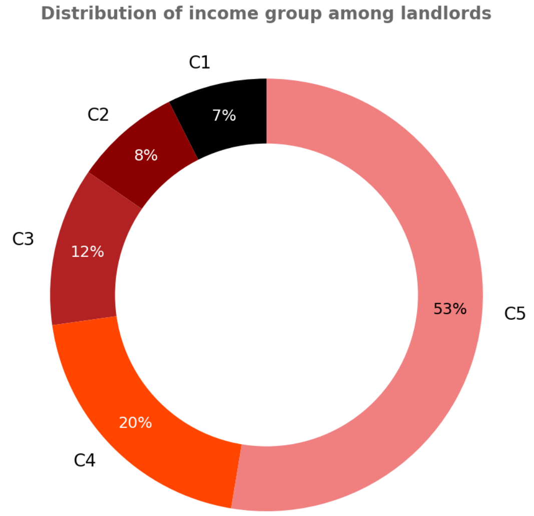


Supplementary Figure 7: **Distribution of income group among landlords in France in 2018.** 'C1' means the first income quintile, i.e. very low income, and 'C5' means the last income quintile, i.e. very high income.

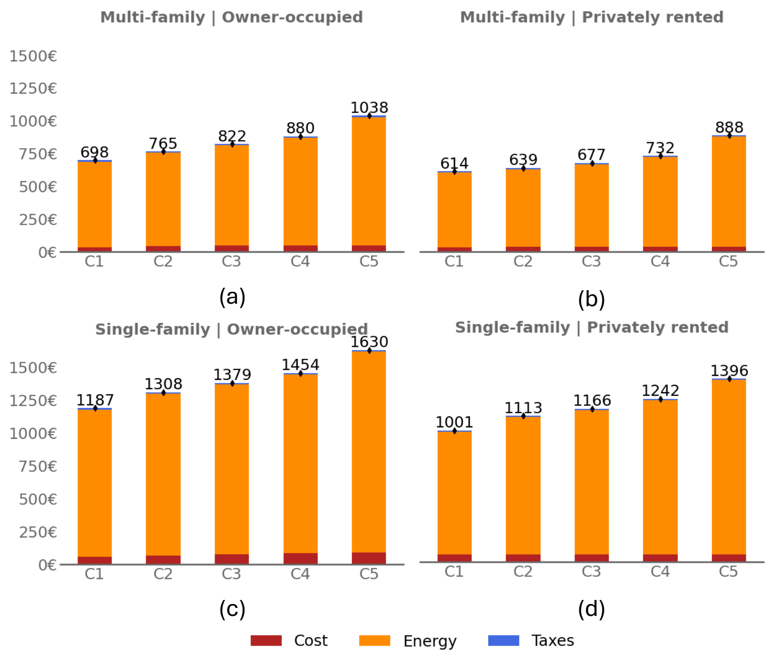


Supplementary Figure 8: **Cost of households in 2018 including energy cost, heater systems investment cost, and taxes due to subsidies.** a. Owner-occupied in multi-family dwellings. b. Privately rented in multi-family dwellings. c. Owner-occupied in single-family dwellings. d. Privately rented in single-family dwellings. 'C1' means the first income quintile, i.e. very low income, and 'C5' means the last income quintile, i.e. very high income.

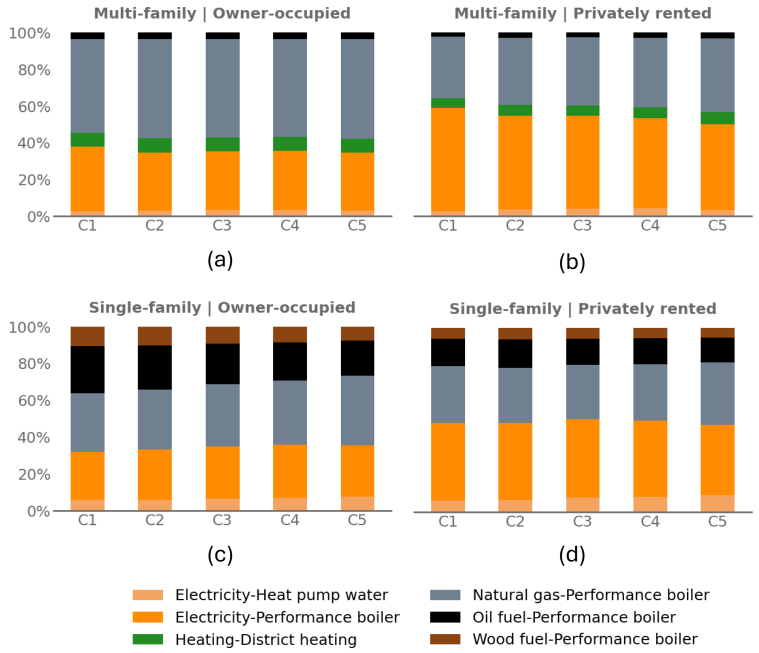


Supplementary Figure 9: **Stock of heating system by household group in 2018.** a. Owner-occupied in multi-family dwellings. b. Privately rented in multi-family dwellings. c. Owner-occupied in single-family dwellings. d. Privately rented in single-family dwellings. ‘C1’ means the first income quintile, i.e. very low income, and ‘C5’ means the last income quintile, i.e. very high income.

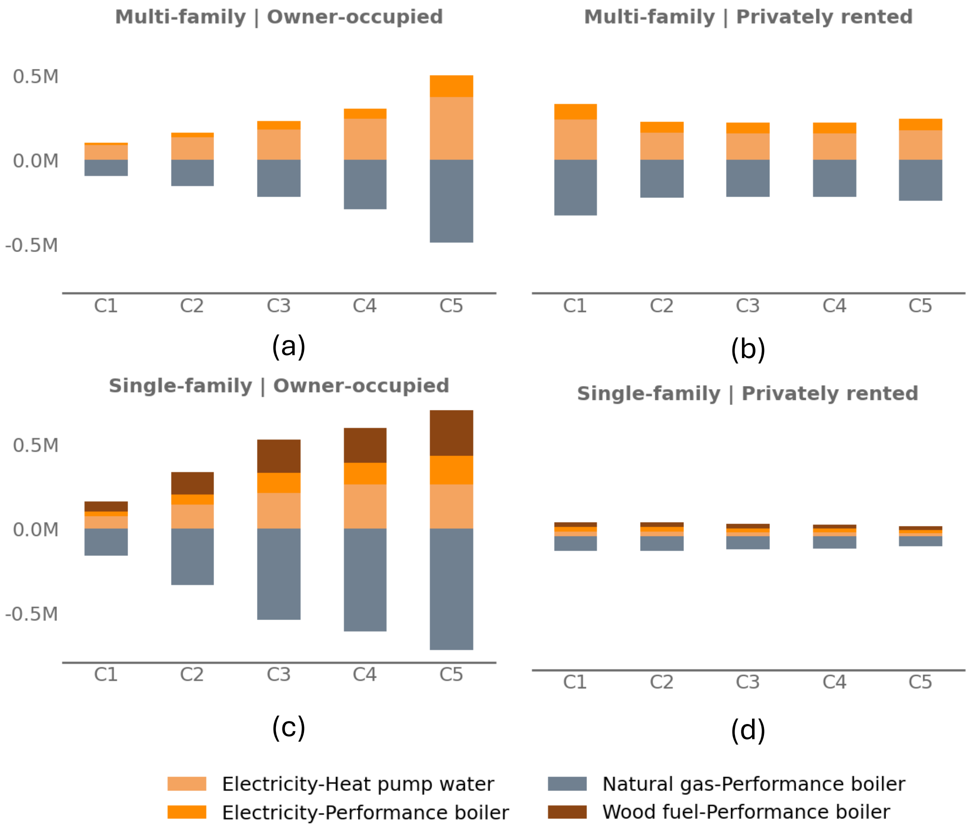


Supplementary Figure 10: **Additional boilers in 2050, if the ban is implemented, in millions of boilers.** a. Owner-occupied in multi-family dwellings. b. Privately rented in multi-family dwellings. c. Owner-occupied in single-family dwellings. d. Privately rented in single-family dwellings. 'C1' means the first income quintile, i.e. very low income, and 'C5' means the last income quintile, i.e. very high income.

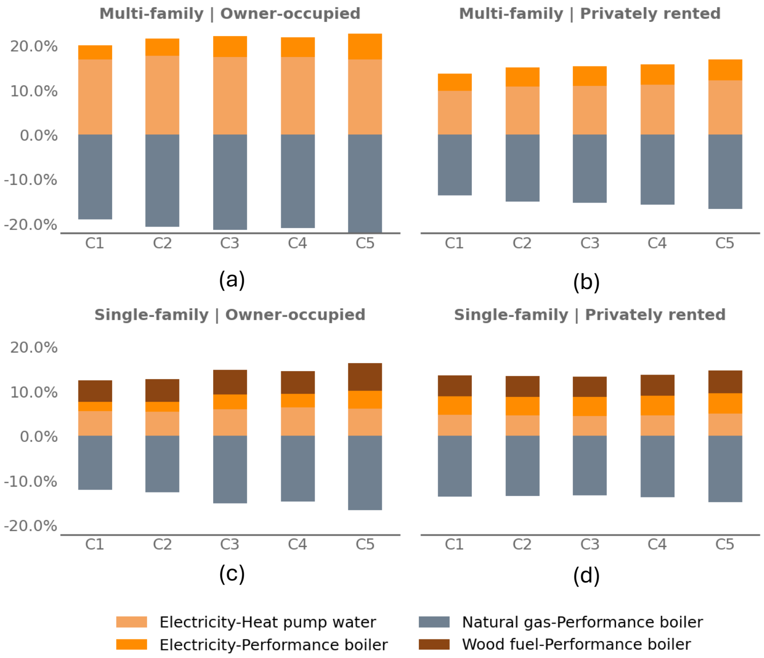


Supplementary Figure 11: **Additional boilers in 2050 if the ban is implemented, as a proportion of total installed boilers in 2050 by household group.** a. Owner-occupied in multi-family dwellings. b. Privately rented in multi-family dwellings. c. Owner-occupied in single-family dwellings. d. Privately rented in single-family dwellings. 'C1' means the first income quintile, i.e. very low income, and 'C5' means the last income quintile, i.e. very high income.

# Supplementary Methods

## Supplementary Method 1

**Total cost incurred by households** The distributional consequences of implementing the ban result from the calculation of the average costs incurred by the household $i$ over time. This cost in time step $t$ includes technology $k$ purchase costs, $\hat{p}^{\mathrm{k}}_{i,t}$ net of subsidies, $s^{\mathrm{k}}_{i,t}$, and energy expenditure $p^{\mathrm{energy}}_{t} \cdot \mathrm{Conso}_{i,t}$, inclusive of taxes meant to cover subsidy costs $T(t, s)$.

We annualized the cost in $t$ by using a 10-year life horizon and a discount rate of 3.9% to mimic household loan terms, as defined in supplementary equation (1).

$$\forall k \in \mathrm{heater}, \mathrm{insulation} \quad p^{\mathrm{k}}_{i,t} = \hat{p}^{\mathrm{k}}_{i,t} / \gamma_{i,t,k,D} \tag{1}$$

Therefore, the $\bar{C}^{\mathrm{investment}}_{I,t}$ paid by households that make investments in $t$ is defined in supplementary equation (2).

$$\bar{C}^{\mathrm{investment}}_{I,t} = \sum_{i \in I} (p^{\mathrm{heater}}_{i,t} - s^{\mathrm{heater}}_{i,t}) \cdot N^{\mathrm{switch}}_{i,t} + (p^{\mathrm{insulation}}_{i,t} - s^{\mathrm{insulation}}_{i,t}) \cdot N^{\mathrm{insulation}}_{i,t} \tag{2}$$

where $N^{\mathrm{switch}}_{i,t}$ is the number of households that buy a new heating system and $N^{\mathrm{insulation}}_{i,t}$ is the number of households that insulate their homes.

We define in supplementary equation (3) $C^{\mathrm{investment}}_{I,t}$ as the sum of cost paid in $t$ that includes past cost that still need to be reimbursed:

$$C^{\mathrm{investment}}_{I,t} = \sum_{tt=t-D}^{t} \bar{C}^{\mathrm{investment}}_{I,tt} \tag{3}$$

The average costs within the group $I$, which contains $N_{I,t}$ households in $t$, are thus defined in supplementary equation (4):

$$C_{I,t} = \frac{C^{\mathrm{investment}}_{I,t} + T(t, s) + \sum_{i \in I} p^{\mathrm{energy}}_{t} \cdot \mathrm{Conso}_{i,t}}{N_{I,t}} \tag{4}$$

The average costs over time is defined in supplementary equation (5).

$$C_I = \frac{\sum_{t=2025}^{2050} C_{I,t} \cdot N_{I,t}}{\sum_{t=2025}^{2050} N_{I,t}} \tag{5}$$

Supplementary Figure 6 show the difference of average total cost for househol group $I$ when the ban is implemented compared to the current policy scenario, which is defined in supplementary equation (6).

$$\Delta C_I = C^{\mathrm{ban}}_{I} - C^{\mathrm{reference}}_{I} \tag{6}$$

Supplementary Table 4: Evolution of CAPEX (€/kWe). New nuclear power can only be installed starting in 2035. Methanation is calculated as the sum of electrolysis CAPEX and Sabatier reaction CAPEX. PV stands for photovoltaic, OCGT stands for open cycle gas turbines, CCGT stands for combined cycle gas turbines.

| Technology | 2025 | 2030 | 2035 | 2040 | 2045 | 2050 | Reference |
|---|---|---|---|---|---|---|---|
| Offshore wind, Floating | 3580 | 3280 | 3130 | 2980 | 2830 | 2680 | RTE[1] |
| Offshore wind, Fixed | 2930 | 2480 | 2380 | 2280 | 2180 | 2080 | RTE[1] |
| Onshore wind, Fixed | 1250 | 1210 | 1190 | 1170 | 1150 | 1130 | RTE[1] |
| Solar PV, ground | 672 | 597 | 557 | 517 | 497 | 477 | RTE[1] |
| Solar PV, Mounted | 967 | 867 | 812 | 757 | 717 | 677 | RTE[1] |
| Nuclear power | NA | NA | 5391 | 5035 | 4505 | 4500 | RTE[1] |
| Methanation | 1700 | 1341 | 1300 | 1274 | 1240 | 1207 | RTE[1] |
| Methanization | 370 | 370 | 370 | 370 | 370 | 370 | ADEME[2] |
| Pyrogazeification | 2500 | 2500 | 2500 | 2500 | 2500 | 2500 | ADEME[2] |
| OCGT | 600 | 600 | 600 | 600 | 600 | 600 | RTE[1] |
| CCGT | 900 | 900 | 900 | 900 | 900 | 900 | RTE[1] |
| CCGT for hydrogen | 1100 | 1100 | 1100 | 1100 | 1100 | 1100 | RTE[1] |

## Supplementary Method 2

**Description of EOLES** The hourly capacity factors for variable renewable energy (VRE) sources, including offshore and onshore wind, as well as solar PV, are defined at the departmental level across France, based on historical data from 2000-2018. Technological cost parameters predominantly derive from the French Transmission System Operator (TSO)'s most recent long-term assessment[1], with additional data from ADEME [2] and Zeyen *et al.* [3] where necessary.

The central scenario for the energy mix incorporates several exogenous assumptions. First, residual electricity demand not endogenously determined by the Res-IRF model — covering uses other than heating — is based on the TSO's central projection of 595 TWh by 2050, factoring in the increased penetration of electric vehicles. Additionally, a demand for 40 TWh of hydrogen by 2050 is anticipated. Second, maximum capacities for VRE and nuclear technologies align with the TSO's central production scenario. Third, the potential for biogas production, through both methanization and pyrogazification processes, is derived from ADEME, adjusted to fit the energy and residential sectors' context.

The main simplification assumptions in the EOLES are consistent with other versions in the EOLES family. First, the power system operates under the copper plate assumption, indicating that electricity produced anywhere in continental France is assumed to be instantaneously available at any consumption point. This assumption treats France as a single node in the model. Second, electricity, methane, and hydrogen demands are considered inelastic. However, due to sector coupling between electricity, methane, and hydrogen networks, demands for electricity in hydrogen production and for gas in electricity generation are elastic and determined endogenously. Third, the model employs linear optimization.

The cost projections for key electricity supply technologies utilized in our simulations primarily derive from RTE[1]. When RTE provides only partial data points between 2025 and 2050, we employ linear extrapolation to estimate the missing values. The annuities are calculated by considering the interest incurred during construction, assuming a uniform discount rate of 3.2% per year. The evolution of Capital Expenditure (CAPEX) is detailed in supplementary table 4, while the evolution of Fixed Operation and Maintenance (FOM) costs is presented in supplementary table 5.

Supplementary Table 5: Evolution of Fixed Operation and Maintenance (FOM) costs (€/kWe/yr). PV stands for photovoltaic, OCGT stands for open cycle gas turbines, CCGT stands for combined cycle gas turbines.

| Technology | 2025 | 2030 | 2035 | 2040 | 2045 | 2050 | Reference |
|---|---|---|---|---|---|---|---|
| Offshore wind, Floating | 95 | 80 | 70 | 60 | 55 | 50.3 | RTE[1] |
| Offshore wind, Fixed | 70 | 58 | 51 | 47 | 41 | 36 | RTE[1] |
| Onshore wind, Fixed | 37.5 | 35 | 32.5 | 30 | 27.5 | 25 | RTE[1] |
| Solar PV, ground | 10.5 | 10 | 9.5 | 9 | 8.5 | 8 | RTE[1] |
| Solar PV, Mounted | 10.5 | 10 | 9.5 | 9 | 8.5 | 8 | RTE[1] |
| Nuclear power | 100 | 100 | 100 | 100 | 100 | 100 | RTE[1] |
| Methanation | 59 | 59 | 59 | 59 | 59 | 59 | RTE[1] |
| Methanization | 37 | 37 | 37 | 37 | 37 | 37 | ADEME[2] |
| Pyrogazeification | 225 | 225 | 225 | 225 | 225 | 225 | ADEME[2] |
| OCGT | 20 | 20 | 20 | 20 | 20 | 20 | RTE[1] |
| CCGT | 40 | 40 | 40 | 40 | 40 | 40 | RTE[1] |
| CCGT for hydrogen | 40 | 40 | 40 | 40 | 40 | 40 | RTE[1] |

Supplementary Table 6: Other constant electricity generation technology parameters. PV stands for photovoltaic, OCGT stands for open cycle gas turbines, CCGT stands for combined cycle gas turbines.

| Technology | Lifetime (yr) | Variable O&M (€/MWh) | Efficiency (%) | Reference |
|---|---|---|---|---|
| Offshore wind, Floating | 40 | 0 | - | RTE[1] |
| Offshore wind, Fixed | 40 | 0 | - | RTE[1] |
| Onshore wind, Fixed | 30 | 0 | - | RTE[1] |
| Solar PV, ground | 30 | 0 | - | RTE[1] |
| Solar PV, Mounted | 30 | 0 | - | RTE[1] |
| Nuclear power | 60 | 6 | - | RTE[1] |
| Methanation | 20 | 5 | 60 | RTE[1] |
| Methanization | 20 | 50 | - | ADEME[2] |
| Pyrogazeification | 20 | 32 | - | ADEME[2] |
| OCGT | 30 | - | 40 | RTE[1] |
| CCGT | 40 | - | 57 | RTE[1] |
| CCGT for hydrogen | 40 | - | 57 | RTE[1] |

Supplementary Table 7: Low-carbon technologies potential in 2050 (GW). PV stands for photovoltaic.

| Technology | 2050 | Reference |
|---|---|---|
| Offshore wind, Floating | 30 | RTE[1] |
| Offshore wind, Fixed | 15 | RTE[1] |
| Onshore wind, Fixed | 58 | RTE[1] |
| Solar pv, Ground | 96 | RTE[1] |
| Solar pv, Mounted | 66 | RTE[1] |
| New nuclear power | 13.5 | RTE[1] |

Supplementary Table 8: Evolution of biogas potential (TWh).

| Potential | Scenario | 2025 | 2030 | 2035 | 2040 | 2045 | 2050 | Reference |
|---|---|---|---|---|---|---|---|---|
| Methanization | S2 | 0 | 14 | 19 | 24 | 29 | 35 | ADEME[2] |
| | S3 | 0 | 19 | 25 | 32 | 39 | 46 | ADEME[2] |
| Pyrogazeification | S2 | 0 | 0 | 0 | 0 | 0 | 0 | ADEME[2] |
| | S3 | 0 | 0 | 5 | 9 | 14 | 19 | ADEME[2] |

The energy system strongly relies on available potential for different technologies, namely biogas (supplementary table 8 and low-carbon technologies (supplementary table 7).

Supplementary Table 9: Evolution of storage CAPEX (€/kWh). PHS stands for pumped hydro storage.

| Technology | 2025 | 2030 | 2035 | 2040 | 2045 | 2050 | Reference |
|---|---|---|---|---|---|---|---|
| PHS | 20 | 20 | 20 | 20 | 20 | 20 | RTE[1] |
| 1h Battery storage | 537 | 439 | 340 | 332 | 324 | 315 | RTE[1] |
| 4h Battery storage | 370 | 299 | 228 | 214 | 200 | 185 | RTE[1] |
| Salt cavern | 350 | 350 | 350 | 350 | 350 | 350 | RTE[1] |

## Supplementary Method 3

**Description of Res-IRF data sources** Supplementary table 11 described all data sources in Res-IRF. Specifically for the case of this study, we recall here the cost assumptions for the heating system, which drive the total cost of the ban on gas boiler.

Supplementary Table 10: Data derived from RTE & ADEME [4]. Cost data includes costs related to domestic hot water systems as part of heating system costs, but do not consider other costs, such as those associated with heat emitters (radiators). Costs are consistent with the JRC DataSet[5] and a previous modeling study[6].

| Heating system | Cost (euro) | Lifetime installation |
|---|---|---|
| Heat pump | 13,000 | 20 |
| Natural gas boiler | 6,000 | 20 |
| Wood boiler | 12,500 | 20 |
| Direct electric | 3,600 | 20 |

Supplementary Table 11: List of data sources used in Res-IRF. * means data are not publicly available.

| Inputs | Source |
|---|---|
| **Energy system** | |
| Energy prices projection | Scenario AME 2021[7] |
| Energy taxes projection | Scenario AME 2021[7] |
| Amount of renewable gas for space heating | Scenario BAU[8] |
| Number of dwelling connected to district heating | Scenario BAU[8] |
| **Housing market** | |
| Demolition rate | Scenario BAU[8] |
| Number of new buildings | Scenario BAU[8] |
| Share of multi-family in new buildings | Scenario BAU[8] |
| Market share heating system construction | Scenario BAU[8] |
| Surface area of new housing | Fidéli (2018) |
| **Macro** | |
| Household income by decile in 2018 | INSEE[9] |
| Income growth | DGEC (2023)* |
| **Initial housing stock** | |
| Housing stock in 2018 | MTE[10]* |
| Building performance characteristics by certificate | ADEME[11] |
| Landlords income | MTE[10] |
| Wood and oil fuel housing | MTE[12] |
| Surface area of dwelling by occupation status | Fidéli (2018)* |
| **Technical data** | |
| U-value of renovated envelope components | ADEME[13] |
| Cost insulation by envelope component | Effinergie[14] |
| Capex heating system | RTE & ADEME[4] |
| Renovation rate | CEE 2017-2018[15] |
| Market share insulation work | TREMI[15] |
| Heating system lifetime | Knobloch et al.[6] |
| Market share heating system | ADEME[8] |
| **Behavioral parameters** | |
| Time preferences discount factor | Stolyarova[16] |
| Status quo bias | Stolyarova[16] |
| Average price elasticity for heat pumps | Own assumption, from Risch[17] |
| **Financing information** | |
| Maximum upfront cost by income class | Dolques et al.[18] |
| Threshold credit constraint | Dolques et al.[18] |
| Average interest rate of households savings | Own assumption |
| Average interest rate of home renovation loan | Dolques et al.[18] |
| **Indicators** | |
| Health cost due to bad housing condition | Dervaux & Rochaix[19] |
| Social discount rate | Ni & Maurice[20] |
| **Thermal module data** | Loga[21] and Arquin et al.[22] |

## Supplementary References


1. RTE. *Futurs énergétiques 2050* fr. Tech. rep. (2022).
2. ADEME. *Mix de gaz 100% renouvelable en 2050 ? [A 100% renewable gas mix in 2050?]* tech. rep. (2018).
3. Zeyen, E., Hagenmeyer, V. & Brown, T. Mitigating heat demand peaks in buildings in a highly renewable European energy system. en. *Energy* **231,** 120784 (Sept. 2021).
4. RTE & ADEME. *Réduction des émissions de CO2, impact sur le système électrique : quelle contribution du chauffage dans les bâtiments à l'horizon 2035 ? [Reducing CO2 emissions, impact on the electricity system: what contribution will heating make to buildings by 2035?]* tech. rep. (Dec. 2020).
5. Hofmeister, M. & Guddat, M. *Techno-economic projections until 2050 for smaller heating and cooling technologies in the residential and tertiary sector in the EU* en. Publisher: European Commission, Joint Research Centre (JRC). Nov. 2017.
6. Knobloch, F. *et al.* FTT:Heat — A simulation model for technological change in the European residential heating sector. en. *Energy Policy* **153,** 112249 (June 2021).
7. MTE. *Synthèse du scénario "Avec mesures existantes" 2021 (AME 2021)* tech. rep. (Ministère de la transition écologique, June 2021).
8. ADEME. *Transition 2050* tech. rep. (2022).
9. INSEE. *Revenu disponible des ménages* May 2021.
10. MTE. *Le parc de logements par classe de consommation énergétique [Housing stock by energy consumption class]* fr. Sept. 2020.
11. ADEME. *DPE Logements (avant juillet 2021)* Mar. 2021.
12. MTE. *Consommation d'énergie par usage du résidentiel* fr. 2018.
13. ADEME, C. *Fiches Bâtiment Résidentiel (BAR).* 2024.
14. Effinergie & ADEME. *Les maisons renovés à basse consommation [Low-energy renovated homes]* tech. rep. (Observatoire BBC, Apr. 2021).
15. MTE. *Enquête sur les travaux de rénovation énergétique dans les maisons individuelles (TREMI)* tech. rep. (Jan. 2020).
16. Stolyarova, E. *Rénovation énergétique de l'habitat en France : analyse microéconométrique du choix des ménages.* These de doctorat (Paris Sciences et Lettres (ComUE), Apr. 2016).
17. Risch, A. Are environmental fiscal incentives effective in inducing energy-saving renovations? An econometric evaluation of the French energy tax credit. en. *Energy Economics* **90,** 104831 (Aug. 2020).
18. Dolques, G., Ledez, M. & Hainaut, H. *Quelles aides publiques pour la rénovation énergétique des logements ?* tech. rep. (I4CE, Feb. 2022).
19. Dervaux, B. & Rochaix, L. *Socio-Economic Evaluation of the Health Effects of Public Investment Projects* tech. rep. (France Stratégie, Mar. 2022).
20. Ni, J. & Maurice, J. *Révision du taux d'actualisation [Revision of the discount rate]* tech. rep. (France Stratégie, Oct. 2021).
21. Loga, T. TABULA Calculation Method – Energy Use for Heating and Domestic Hot Water. en, 56 (2013).
22. Arquin, C., Parc, J., Daunay, J. & Tazi, A. *À quelles conditions le secteur résidentiel peut-il atteindre la neutralité carbone telle que définie dans la SNBC ?* fr. Tech. rep. (Pouget Consultants, Carbone 4, Jan. 2020).
